# Coupling Agents in Acoustofluidics: Mechanisms, Materials, and Applications

**DOI:** 10.3390/mi16070823

**Published:** 2025-07-19

**Authors:** Shenhao Deng, Yiting Yang, Menghui Huang, Cheyu Wang, Enze Guo, Jingui Qian, Joshua E.-Y. Lee

**Affiliations:** 1 Anhui Province Key Laboratory of Measuring Theory and Precision Instrument, School of Instrument Science and Opto-Electronics Engineering, Hefei University of Technology, Hefei 230009, China; 2 School of Microelectronics, Hefei University of Technology, Hefei 230009, China; 3 Centre for Audio, Acoustics and Vibration, University of Technology Sydney, Ultimo, NSW 2007, Australia

**Keywords:** acoustofluidics, acoustic coupling agents, acoustic impedance matching, microfluidic devices, coupling agents, biomedical applications

## Abstract

Acoustic coupling agents serve as critical interfacial materials connecting piezoelectric transducers with microfluidic chips in acoustofluidic systems. Their performance directly impacts acoustic wave transmission efficiency, device reusability, and reliability in biomedical applications. Considering the rapidly growing body of research in the field of acoustic microfluidics, this review aims to serve as an all-in-one reference on the role of acoustic coupling agents and relevant considerations pertinent to acoustofluidic devices for anyone working in or seeking to enter the field of disposable acoustofluidic devices. To this end, this review seeks to summarize and categorize key aspects of acoustic couplants in the implementation of acoustofluidic devices by examining their underlying physical mechanisms, material classifications, and core applications of coupling agents in acoustofluidics. Gel-based coupling agents are particularly favored for their long-term stability, high coupling efficiency, and ease of preparation, making them integral to acoustic flow control applications. In practice, coupling agents facilitate microparticle trapping, droplet manipulation, and biosample sorting through acoustic impedance matching and wave mode conversion (e.g., Rayleigh-to-Lamb waves). Their thickness and acoustic properties (sound velocity, attenuation coefficient) further modulate sound field distribution to optimize acoustic radiation forces and thermal effects. However, challenges remain regarding stability (evaporation, thermal degradation) and chip compatibility. Further aspects of research into gel-based agents requiring attention include multilayer coupled designs, dynamic thickness control, and enhancing biocompatibility to advance acoustofluidic technologies in point-of-care diagnostics and high-throughput analysis.

## 1. Introduction

The 1990s witnessed the transition of microfluidics from theory to practice, marked by a pivotal breakthrough: the conceptualization of the “lab-on-a-chip.” Scientists envisioned integrating complex laboratory-scale chemical analyses into miniature chips to achieve automated, miniaturized, and high-efficiency operations, thereby reducing reagent/sample consumption, enabling rapid turnaround times, and permitting precise microenvironment control. With the advent of the 21st century, microfluidics entered a phase of rapid expansion, fueled by interdisciplinary applications that established it as a cornerstone platform for innovations in chemical analysis, biology, medicine [1,2,3,4,5,6,7,8,9], and environmental monitoring [10].

Acoustofluidics has emerged as a prominent technique for microscale manipulation, alongside magnetic [11,12], optical [13], and dielectrophoretic (DEP) [14] methods. By converting electrical signals into acoustic waves (SAWs [15,16,17,18,19,20] or BAWs [21,22,23,24,25]) via piezoelectric transducers, acoustofluidics enables label-free, non-contact particle/cell control with inherent biocompatibility [10,18,26], low power consumption [27], and high throughput [17]. Its wide range of enabling functionalities encompasses particle concentration [18], patterning [28,29,30], sorting [17], aerosolization [31], droplet actuation [28], and biochemical analysis [32,33]. With such versatility, acoustofluidic devices are positioned to become a transformative platform for biomedical diagnostics and lab-on-a-chip systems.

However, conventional SAW-based acoustofluidic devices face two critical limitations as follows:

1. **Cost inefficiency**: Single-use substrates (e.g., lithium niobate) are prohibitively expensive, hindering applications in contamination-sensitive analyses [20];
2. **Design constraints**: Direct bonding of microchannels to piezoelectric substrates risks cross-contamination, cell loss, and structural deformation [34].

To address these challenges, researchers have developed detachable, reusable acoustofluidic devices, where a single transducer can interface reversibly with multiple microfluidic chips (referred to as “superstrates” [32,35,36,37,38]). This approach significantly reduces costs while maintaining biocompatibility through disposable chips.

A key enabler of such systems is the acoustic coupling agent—a deformable interfacial material that mitigates energy loss due to imperfect contact between rigid layers. Coupling agents (e.g., liquids like water [35,36] or glycerol [35], gels like KY jelly [18], or polymers like PDMS [17,20]) facilitate efficient wave transmission from transducers to microchannels. While liquid agents offer high acoustic impedance matching and flexibility, they suffer from evaporation and instability. Gel-based agents improve stability but exhibit high acoustic attenuation. Poly(dimethylsiloxane) (PDMS) has emerged as a promising alternative due to its flexibility, chemical stability, biocompatibility, and optical transparency, enabling enhanced interfacial adaptability and long-term performance.

Despite extensive reviews on SAW microfluidics, a systematic analysis of coupling agents—linking material design, acoustofluidic mechanisms, and applications—remains lacking. This review bridges this gap by categorizing reported coupling agents, elucidating the physical mechanisms that support acoustofluidic functions, evaluating the applications they enable, and identifying challenges to guide the development of next-generation acoustofluidic devices from the perspective of gel-based coupling agents.

## 2. Overview of Acoustic Coupling Agents

Imperfect contact between rigid materials leads to inefficient acoustic energy transfer from the acoustic transducers (forming the substrate) to the overlaying superstrate. This necessitates the use of deformable interfacial materials known as acoustic coupling agents. These coupling layers must exhibit key characteristics, including acoustic impedance matching, stability, compatibility, and ease of fabrication. Their primary function is to minimize acoustic wave reflection (back into the substrate) and energy loss at interfaces, thereby enhancing the efficiency of coupling acoustic energy from the acoustic actuator to the static microfluidic chip, thereby lowering power consumption.

In typical configurations, symmetric surface acoustic waves propagate through the coupling layer from the piezoelectric substrate to a glass cover layer, where they excite bulk vibrations in the form of symmetric Lamb waves. While Lamb waves demonstrate reduced acoustic intensity compared to surface acoustic waves, they remain sufficiently strong to induce acoustic streaming within droplets. Acoustofluidic devices commonly employ various coupling media, categorized as either liquid-based (aqueous or oil-based) or non-liquid (gel-based or polymeric) agents. This section systematically examines the physical principles underlying the use of acoustic interface materials in acoustofluidic devices, followed by a classification of common materials with respect to their properties, advantages, and limitations.

### 2.1. Physical Principles of Acoustic Coupling Agents

The fundamental role of acoustic interface materials in acoustofluidic systems is to establish efficient acoustic transmission pathways, operating through three primary mechanisms: impedance matching, signal attenuation reduction, and interfacial contact optimization.

#### 2.1.1. Acoustic Impedance Matching: Minimizing Interface Reflection

Acoustic impedance (Z), defined as follows:

(1) $$\;Z\;=\rho v_{p}$$

where ρ is density and $v_{p}$ is phase velocity, which represents a material’s resistance to acoustic wave propagation and determines energy transfer efficiency between media. The reflection coefficient (R) at the interface between two materials characterized by impedances Z_2_ and Z_1_ is defined by [39] the following:

(2) $$R=[\frac{Z_{2}-Z_{1}}{Z_{2}+Z_{1}}{]}^{2}$$

For instance, the extreme impedance mismatch between air (400 Rayl) and biological tissue (1.63 MRayl) leads to 99.998% energy reflection. Coupling agents bridge this gap by providing intermediate impedance values. For example, aqueous gels (1.45–1.60 MRayl) that closely match tissue impedance (1.5–1.7 MRayl) can reduce reflections to negligible levels. Taking the field of acoustic sensors as an illustration, experimental evidence shows that ultrasonic gels improve sensor signal reception by 250-fold compared to air interfaces [39].

#### 2.1.2. Attenuation Mitigation: Reducing Propagation Losses

Apart from reflections at the interface, another factor for reducing transmission efficiency is propagation losses through the media between the superstrate and substrate. Acoustic energy attenuation arises from absorption and scattering (caused by structural discontinuities), for which the attenuation coefficient is given by the following:

(3) $$\alpha =\frac{\omega^{2}\eta }{2v_{p}}$$

where ω denotes the propagating angular frequency and η is the viscosity of the media. Ideal coupling agents exhibit low attenuation characteristics, with water (0.0022 dB/cm·MHz) and gels (0.05 dB/cm·MHz) providing substantially lower attenuation compared to air (7.5 dB/cm·MHz) [39].

#### 2.1.3. Interfacial Contact Optimization

The mechanical properties of coupling agents critically influence interfacial contact quality. Liquid/gel agents achieve conformal contact by filling microscopic surface irregularities, eliminating air gaps that cause acoustic shorting. KY jelly, for example, enables reversible SAW device chip connections through viscoelastic adhesion, maintaining stable coupling at RF powers up to 38 dBm while allowing non-destructive disassembly [18]. Optimal thickness control (20–30 μm) balances coupling efficiency with mechanical stability.

Through these synergistic mechanisms (impedance matching, attenuation control, and contact optimization), couplants critically determine the principal energy loss pathways in acoustofluidic systems. Material innovations continue to expand their capabilities across diverse applications.

### 2.2. Physical Characteristics and Classification of Acoustic Coupling Agents

#### 2.2.1. Liquid-Based Couplants (Aqueous/Oil)

**Physical Characteristics:**

Water represents the simplest coupling medium due to its high transmission coefficient and low reflection coefficient. The operational duration of aqueous couplants can be extended by incorporating hydrophilic compounds (e.g., glycerol) to retard evaporation. These liquid agents demonstrate excellent droplet handling capabilities and provide sufficient acoustic coupling efficiency while offering advantages in preparation simplicity and wettability; these require only placement between the sample and transducer. Prior studies have documented methods for measuring glycerol concentration and particle liquid concentration [40,41]. The acoustic properties of typical liquid-based couplants are summarized in Table 1 below.

**Advantages and Disadvantages:**

**Figure 1:**
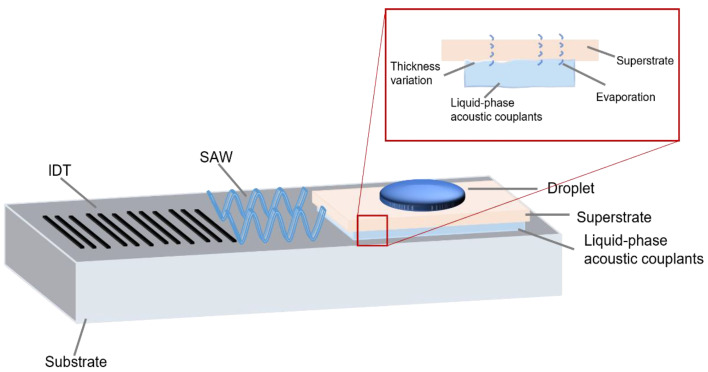
Instability of aqueous and oil-based coupling agents.

Aqueous and oil-based coupling agents are widely employed due to their ease of preparation and application, particularly in scenarios where their acoustic energy attenuation effects are negligible. In short-term experiments, their high transmission coefficients and low cost make them preferable, with minimal consideration of adverse effects. However, in prolonged experiments, their tendency to evaporate or aerosolize under increasing acoustic power cannot be ignored, as shown in Figure 1, necessitating periodic reapplication for continued operation. While the risks of displacing the superstrate, associated with aqueous couplants, are often mitigated by incorporating oil components, this adjustment significantly increases acoustic energy attenuation. Furthermore, potential contamination effects of the additives during long-term usage need to be carefully considered.

#### 2.2.2. Gel-Based Coupling Agents

**Physical Characteristics:**

Common gel-based coupling agents include agarose gel, sodium alginate gel, and collagen gel. In microacoustic fluidic applications, KY gel is frequently selected due to its semi-solid properties and aqueous composition. Its primary components are water, glycerol, and minor thickeners, offering long-term stability and high coupling efficiency compared to pure liquids, while maintaining biocompatibility. However, it exhibits strong adhesion, making the separation of the superstrate and substrate after intended use challenging.

**Advantages and Limitations:**

**Figure 2:**
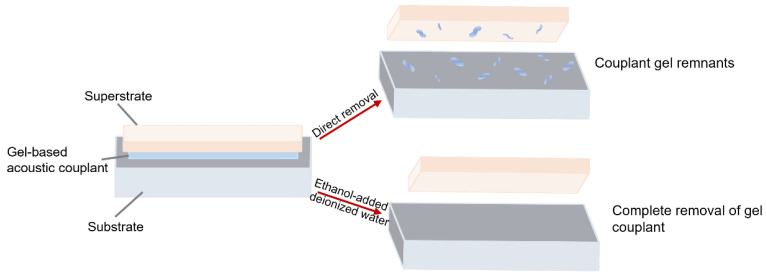
Surface differences before and after using deionized water and ethanol.

KY gel demonstrates superior long-term stability, effective crosslinking, biocompatibility, and non-toxicity. Its semi-solid nature provides strong adhesion and high acoustic coupling efficiency. But these advantages also simultaneously complicate the implementation of disposable devices due to the difficulty of detaching from solid interfaces. Post-experiment removal via repeated washing is impractical, and residual contamination may interfere with the results. To address this, deionized water and ethanol are typically used to dissolve KY gel without damaging the substrate [10] (Figure 2), facilitating device reuse.

#### 2.2.3. Polymer-Based Coupling Agents

**Physical Characteristics:**

Polymer-based coupling agents include epoxy resins, silicones, conductive adhesives, and polyurethanes. In microacoustic fluidics, the most commonly used polymer coupling agents are UV–curable epoxy resins and polydimethylsiloxane (PDMS). The acoustic properties of PDMS are dependent on the film thickness. At room temperature, PDMS demonstrates a density of 1030 ± 0.7 kg/m^3^ [43]. As demonstrated in [44], particle migration velocity decreases from 115.3 μm/s (for 2 mm thick PDMS) to 56.7 μm/s (for 6 mm thick PDMS). Additionally, surface acoustic wave (SAW) propagation in PDMS shows a linear attenuation coefficient of −5 dB/mm, as characterized in [45]. The acoustic properties of typical polymer materials are summarized in Table 2.

UV–curable epoxy resin and polydimethylsiloxane (PDMS) are widely employed as polymer-based coupling agents in microacoustic fluidic applications.

**UV–Curable Epoxy Resin:**

UV epoxy resin is a photo-polymerizable material, typically composed of epoxy monomers, photoinitiators, and additives. Upon ultraviolet (UV) irradiation, the photoinitiator triggers a polymerization reaction, converting the resin from a liquid to a solid state. Prior to curing, its liquid form allows for easy coating and molding. Once cured, it exhibits high mechanical strength, chemical resistance, and thermal stability, facilitating both experimental procedures and post-experiment device handling.

**Polydimethylsiloxane (PDMS):**

PDMS is a silicone-based elastomer, typically formed by mixing a prepolymer (base) with a crosslinking agent (curing agent). The mixing ratio of these components determines their mechanical properties, offering tunable compliance and dimensional stability, which simplifies fabrication. However, as a soft material, PDMS exhibits a high acoustic attenuation coefficient, leading to the partial absorption of Rayleigh wave energy on LN substrates.

**Advantages and Disadvantages:**

**Figure 3:**
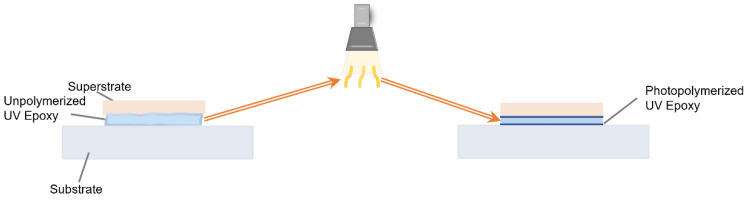
Effects of ultraviolet (UV) light on UV–curable epoxy resin states.

UV epoxy resin and PDMS are commonly used polymer coupling agents in modern acoustic microfluidics, featuring different mechanical properties due to their distinct forms. Under the influence of varying ultraviolet light intensities, the physical form of UV epoxy resin changes (Figure 3), and it is often applied as a liquid to cover surfaces perfectly and minimize gaps, ensuring more uniform and less deviated acoustic energy transmission in subsequent steps. After curing, the UV epoxy resin enhances adhesion, firmly bonding the devices on both sides of the coupling layer. Subsequent removal of the resin can be carried out based on its different physical properties. However, the acoustic energy transmission efficiency of UV epoxy resin is significantly lower than that of water-based and oil-based coupling agents during short-term usage, attributed to its performance degradation over time [47], which requires special attention in acoustic microfluidic applications that demand high-power acoustic energy transmission, which will be discussed in more detail later in the section on applications.

The mechanical and acoustic properties of PDMS vary significantly with its formulation. Soft PDMS exhibits higher acoustic attenuation and faces fabrication challenges due to its viscous nature and edge protrusion during thin-film production. Experimental studies by Genovés et al. [43] demonstrate that stiff PDMS exhibits an acoustic attenuation of 200–400 m^−1^ within the 3–7 MHz frequency range, with only marginal temperature dependence observed between 20 and 50 °C. In contrast, soft PDMS shows significantly lower attenuation overall. The attenuation characteristics reveal material-specific dependencies: stiff PDMS follows a pronounced power-law relationship with increasing frequency, whereas soft PDMS displays markedly weaker frequency dependence. These differences likely stem from distinct viscoelastic properties governed by crosslinking density.

In contrast, hard PDMS demonstrates lower attenuation and enables ultrathin films that enhance energy coupling efficiency between SAW devices and silicon substrates. However, fabrication limitations constrain achievable thinness, necessitating a compromise between attainable energy transfer performance and practical thickness.

A key challenge arises from heat generation, where the relationship between enhanced coupling efficiency and heat production is non-trivial. Its impact is jointly governed by energy transmission efficiency, device power regulation, and the underlying mechanisms of thermal effect induction, and it is particularly pronounced in high-power surface acoustic wave (SAW) device applications. Excessive heat can disrupt experiments and complicate theoretical modeling, rendering this interplay a critical focus of inquiry.

Specifically, heat generation mechanisms in SAW devices are multifaceted. They encompass Joule heating from resistive losses in interdigital transducers (IDTs), as well as dielectric loss-induced heating arising from substrate polarization. In the context of interactions with biological tissues, heat generation in PDMS couplants primarily stems from mechanisms such as viscous dissipation and acoustic attenuation during acoustic energy propagation.

## 3. Basic Applications of Acoustic Coupling Agents in Acoustic Microfluidics

In this section, we review some fundamental applications of acoustic coupling agents in acoustic microfluidic platforms (particularly gel-based coupling agents): (1) achieving efficient acoustic signal transmission and mode conversion, (2) ensuring stable interface connection and device reusability, and (3) enabling acoustic field modulation. These applications have become key aspects in detachable and reusable acoustic microfluidic platforms.

### 3.1. Efficient Acoustic Signal Transmission and Mode Conversion

**Figure 4:**
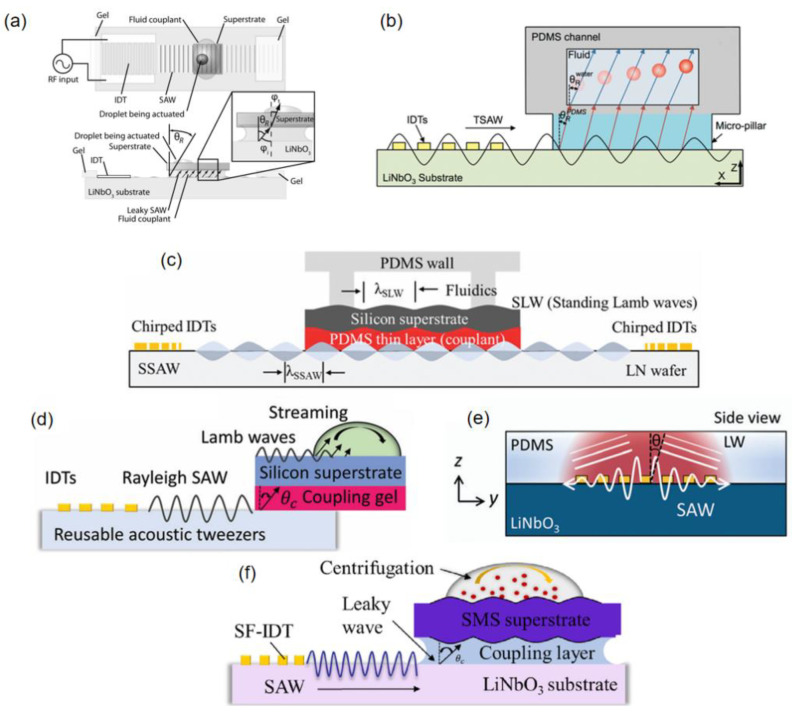
(**a**) Schematic diagram of fluid coupling, illustrating the coupling of leaky surface acoustic wave (SAW) radiation to the fluid in the upper substrate containing microfluidic devices (top and side views). (**b**) Wave propagation from the SAW transducer to the fluid channel. (**c**) Cross-sectional view showing the leakage of SSAW from the LN substrate through the coupling layer as a standing Lamb wave to the silicon substrate. (**d**) Cross-sectional view of the wave propagation process, including leakage, coupling, conversion, and final incidence into the water droplet. (**e**) Side view (yz-plane) of acoustic wave refraction as a longitudinal wave into the PDMS membrane, with acoustic-to-thermal energy conversion. (**f**) Cross-sectional schematic of acoustic wave propagation, highlighting the final reduction in acoustofluidic energy upon refraction in the water droplet. Reprinted with permission from [17,18,20,35,48,49].

Acoustic wave transmission media serve as critical interfaces between surface acoustic wave (SAW) devices and superstrates, enabling efficient energy transfer and mode conversion for particle manipulation. As shown in Figure 4a, Rayleigh waves generated by SAW devices propagate through the coupling agent (e.g., PDMS [17]) to silicon substrates, converting into Lamb waves that drive fluidic acoustic radiation forces (ARFs) for particle capture [35].

In the detachable system by Ma et al. [17], Rayleigh-to-Lamb wave conversion follows angular relationships derived from material sound velocities. In the case of the interface between PDMS (acoustic velocity c = 1019 m/s) and LN (acoustic velocity $c_{LN}=3978\;m/s$), the respective Rayleigh angle is $\theta_{R}^{PDMS}\approx 14.8{}^{\circ}$ $\left(\theta_{R}^{PDMS}={sin}^{-1}(\frac{c_{PDMS}}{c_{LN}})\right)$. Subsequent refraction into water (acoustic velocity $c_{water}=1495\;m/s$) above the superstrate yields a different Raleigh angle $\theta_{R}^{water}\approx 22.1{}^{\circ}$ (Figure 4b). These angle-dependent transmissions ensure controlled wave propagation and fluid actuation, underpinning precise acoustofluidic operations.

As shown in Figure 4c, Qian et al. demonstrated dual-chip acoustofluidic control using a 35 μm PDMS coupling layer to transfer Rayleigh waves from an LN SAW device to a silicon-based chip (400 μm thick), generating standing Lamb waves (SLWs) for microparticle trapping [20]. The SLW pressure nodes—controlled by adjusting the frequency (13.3–19.95 MHz)—enable precise particle manipulation by optimizing the PDMS coupling agent thickness to minimize acoustic loss.

Figure 4f illustrates the abovementioned plug-and-play acoustic tweezers [48], where a coupling agent converts SAW-induced Rayleigh waves into Lamb/Rayleigh waves in the superstrate. This generated droplet-internal vortices via wave refraction, enabling particle concentration and separation through acoustofluidic streaming.

KY gel (thickness 20–30 μm) serves as an elastic medium. In Qian’s dual-chip acoustic flow control device with a periodic patterned silicon upper layer coating [18], the Rayleigh waves generated by the SAW device (LiNbO_3_ substrate) are leaked to the upper silicon chip at a critical angle (θ_c_ = 22.18°), as shown in Figure 4d. This excites Lamb waves and forms an asymmetric acoustic flow field within the droplet, which drives the particles in the droplet to rotate and concentrate towards the center of the droplet. This acoustic wave mode conversion relies on the acoustic impedance matching characteristics of the coupling agent. For instance, the acoustic attenuation coefficient of KY gel is relatively low, allowing the acoustic wave energy to effectively penetrate the interface. The scattering effect of the periodic structure on the acoustic wave enhances the acoustic streaming force (ASF). The ASF is the drag force generated by acoustic streaming, which is the periodic fluid motion induced by sound waves propagating through the fluid. Here, the ASF creates a stagnation point of minimum fluid velocity at the droplet center where particles accumulate. In conjunction, acoustic radiation force (ARF) acts directly on the same particles to trap them through acoustic forces.

**Figure 5:**
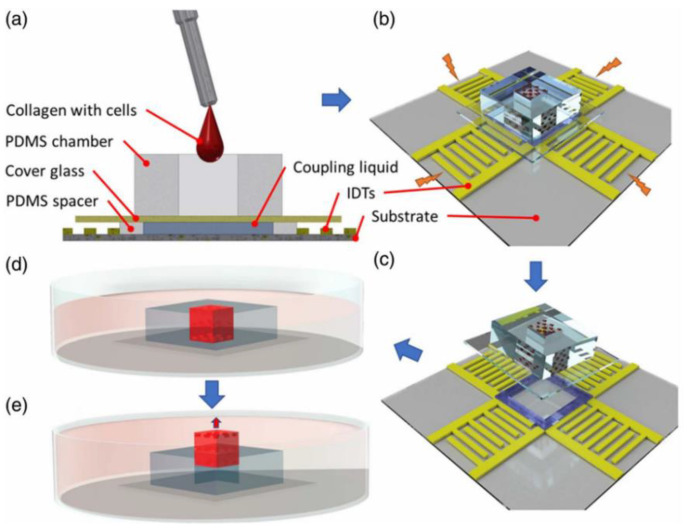
Experimental setup and procedure schematic. (**a**) Inject the hydrogel–cell mixture into an open-top PDMS glass chamber that has been pre-placed on the SAW device. Coupling liquid fills the gap between the chamber and the device, with a PDMS spacer preventing leakage. (**b**) Activate the interdigital transducers (IDTs) of the SAW device to initiate 3D patterning. (**c**) Separate the chamber from the SAW device. (**d**) Transfer the chamber to a Petri dish with culture medium. (**e**) Use buoyancy-driven lift to immerse the 3D polymerized hydrogel construct in the culture medium and release it from the Petri dish. Adapted with permission from [34].

Nguyen et al. [34] employed a 70% ethanol solution as an acoustic coupling agent in a reusable acoustofluidic device for patterning particles and breast cancer cells within a 3D scaffold (Figure 5a). The ethanol’s optimal acoustic impedance enabled efficient surface acoustic wave (SAW) transmission from the LN substrate to the PDMS glass chamber. Here, Rayleigh waves leaked through the coupling layer, exciting Lamb waves that created periodic pressure nodes in the hydrogel. These nodes directed particles/cells into organized 3D crystalline patterns (e.g., within a 5 × 5 × 5 mm^3^ chamber). A PDMS spacer-controlled coupling layer thickness optimized wave transmission at 24.85 dBm, minimizing energy loss while maintaining stable pressure fields unaffected by thermal convection.

The acoustic coupling agent can efficiently transmit the surface acoustic waves (SAWs) generated by piezoelectric substrates (e.g., LN) to the upper substrate while also converting them into suitable acoustic wave modes (such as Lamb waves) for microfluidic operations. In the asymmetric acoustic wave-driven acoustic flow control technology by Zheng et al. [50], the coupling agent acts as an acoustic wave guiding medium, enabling the conversion of SAWs from the symmetric mode of linear propagation to asymmetric Lamb waves. These latter waves are excited in the upper glass substrate and trigger acoustic flow effects, promoting the mixing of substances within droplets and the regulation of crystal growth.

Couplants facilitate efficient wave transmission between piezoelectric transducers (e.g., PZT) and microfluidic systems. In Qian et al.’s platform [51], a 15 µm UV adhesive optimizes bulk acoustic wave (BAW) transmission via impedance matching (silicon: 21.37 MRayl; PZT: 3791 m/s), while sealing layer materials dictate wave behavior. PDMS (1.05 MRayl) creates localized potential wells via impedance mismatch, whereas glass (14.42 MRayl) enhances transmission for distinct particle patterns.

These agents also act as energy bridges, exemplified in Park et al.’s thermoacoustic tweezers [49], where a PDMS film converts SAWs into longitudinal waves (LWs) and harnesses viscoelastic damping to generate localized heating (Figure 4e). This dual functionality—wave transmission and energy conversion—enables precise droplet manipulation in disposable chips while balancing thermal and acoustic energy utilization.

Gel-based coupling agents, due to their generally low acoustic attenuation coefficients, help acoustic wave energy effectively penetrate the interface, provide adequate acoustic impedance matching, and provide a guarantee for the manipulation of particles by acoustic energy. Therefore, gel-based couplants often become the top choice when selecting acoustic couplants.

### 3.2. Stable Interfacial Connection and Device Reusability

Effective acoustic coupling between surface acoustic wave (SAW) devices and superstrates requires a tight interfacial contact to minimize impedance mismatch and energy loss. Coupling agents enhance wave transmission, mode conversion, and reliable bonding while enabling reversible device assembly.

Nguyen et al. [34] demonstrated this using ethanol as a coupling agent in a reusable acoustofluidic system (Figure 5c). The ethanol-mediated physical adsorption allowed rapid PDMS glass chamber replacement via an open-top design while maintaining acoustic integrity. Post-use, buoyancy-driven detachment (Figure 5e) preserved both the SAW device and chamber for reuse. Ethanol’s easy-clean properties further streamlined maintenance, making the system ideal for high-throughput applications requiring frequent chamber exchanges.

Compared with traditional coupling agents (such as water), high-viscosity gel-type coupling agents (such as KY gel) can significantly improve the coupling stability during long-term operation. Experiments show [32] that when water is used as the coupling agent, due to rapid evaporation, the coupling efficiency drops by 75% after 8 min, and thus it is inapplicable to experiments operating for more than 10 min. In contrast, KY glue, with its aqueous gel characteristics, maintains a stable coupling efficiency for over 25 min, and the experimental results of Qian et al. on the coupling efficiency of different coupling agents over time [48] provide strong evidence for this view.

To address the problem of interface degradation under high-power input, the coupling agent needs to have anti-evaporation and long-term stability characteristics. In the plug-and-play device developed by Qian [48], the effects of four coupling agents, including DI water, ethanol, KY gel, and UV epoxy resin, were compared. The transmission efficiency of DI water, ethanol, and KY gel was similar and was significantly higher than that of UV epoxy resin. Deionized water and ethanol were effective in short-time, low-power experiments but were prone to evaporation under high power, resulting in a decrease in coupling efficiency. In contrast, KY gel, due to its high viscosity and low volatility, remained stable during long-term operation of over 10 min, making it the preferred coupling agent for high-power input requirements.

**Figure 6:**
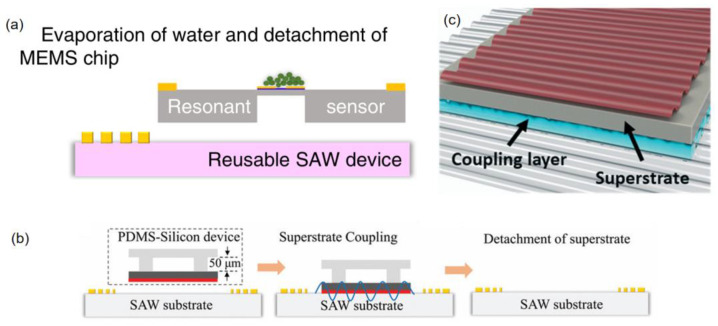
(**a**) Micro-object deposition and measurement: droplet evaporates in seconds and leaves microparticles on the TPoS resonant sensor (fabricated in the MEMS chip), which measures the weight of the particles. Prior to electrical measurement of the chip, the MEMS is non-destructively detached from the LN substrate. (**b**) Cross-sectional schematic of silicon superstrate attachment/detachment via the PDMS coupling layer to preserve SAW device integrity. (**c**) Acoustic energy transfer from the reusable transducer via superstrate coupling layers. Adapted with permission from [10,20,52].

Acoustic coupling agents enable reversible, contamination-free interfaces between piezoelectric substrates and disposable microfluidic components. In Qian et al.’s resonant sensor [10], a hydrophobic MEMS chip (103.4° contact angle) and colloidal coupling agent maintained a spherical 4 μL droplet for precise particle positioning, with post-experiment dissolution (water/ethanol, Figure 6a) enabling non-destructive disassembly in 30 s. While liquid agents (5–20 μm layers) face scattering and stability issues [50], PDMS films excel with thermal stability (7–8 °C rise under RF power) and reusable adhesion (>50 cycles via van der Waals forces) [50], as demonstrated in Qian’s dual-chip system [20] (ethanol detachment, Figure 6b) and Kolesnik et al.’s hard PDMS interfaces [52] (Figure 6c).

As seen in the literature, gel-based agents dominate acoustofluidic systems due to their operational stability and manufacturability, while elastic polymers/liquids balance performance and cost by decoupling expensive transducers from disposable chips. These innovations together support high-throughput applications requiring frequent component replacement without compromising precision or biocompatibility.

### 3.3. Acoustic Field Modulation

Beyond enabling efficient energy transfer and stable interfacial connections, acoustic impedance matching layers play a pivotal role in precision acoustic field modulation through tailored material properties. This functionality expands the manipulation capabilities of acoustofluidic systems.

The thickness of the couplant affects acoustic field distribution and energy transfer efficiency. Experimental investigations conducted by Hodgson et al. have shown that, at an input power of 350 mW, the droplet velocity increased from 2 mm/s to 6 mm/s when the couplant thickness was increased from 7.14 μm to 28.6 μm [35]. A thicker fluid coupling layer (ranging from 7.14 μm to 28.6 μm) can enhance the transmission of acoustic energy to the superstrate by having this thickness close to a quarter-wavelength of the acoustic waves in the fluid (approximately 18.5 μm for 20 MHz acoustic waves in water) to create a standing wave enhancement effect. This effect optimizes acoustic impedance matching, reduces reflections at the LN–fluid and fluid–glass interfaces, and enhances energy transmissivity.

Meanwhile, a thicker coupling layer elongates the propagation path of acoustic waves in the fluid, thereby bringing the incident angle closer to the critical angle ($\theta_{R}\approx 23{}^{\circ}$), strengthening the acoustic pressure field at the fluid–glass interface, and facilitating the excitation of high-order asymmetric Lamb waves. Such waves enable efficient energy transmission within the thin glass superstrate. The experimental findings obtained via scanning laser Doppler vibrometry reveal that the displacement amplitude of the glass surface attains 50% of that of the original surface acoustic wave (SAW) on the piezoelectric substrate, indicating effective energy transfer. Furthermore, a thicker coupling layer (14–28 μm) is capable of reducing interface reflections and viscous dissipation in the fluid. Experimental data indicate that, at an input power of 350 mW, the droplet velocity exhibits a significant increase with increasing coupling layer thickness (e.g., the velocity of 3 μL water droplets increases from approximately 1 mm/s to greater than 6 mm/s). The fourth power of the droplet velocity exhibits a linear correlation with the input power. However, when the thickness exceeds 30 μm, efficiency diminishes due to viscous losses and modal mismatch.

A key advantage of this mechanism is that the superstrate can be independently fabricated at low cost, while the piezoelectric substrate can be reused repeatedly. By optimizing impedance matching and promoting Lamb wave excitation, more radio frequency (RF) acoustic energy is converted into mechanical waves within the superstrate, thereby driving microfluidic motion. This thus confirms that a thicker couplant layer can enhance energy transfer. Such thickness-dependent regulation enables the precise optimization of droplet manipulation parameters. Additionally, the acoustic impedance matching between the couplant and the substrate dictates wave behavior: the significant impedance mismatch between glass (with a sound velocity of 5100 m/s) and water (with a sound velocity of 1482 m/s) triggers Lamb wave conversion and induces out-of-plane vibrations. The resulting displacement amounts to approximately half of that of the original SAW on the LN substrate, while still retaining sufficient energy to achieve effective microfluidic actuation.

**Figure 7:**
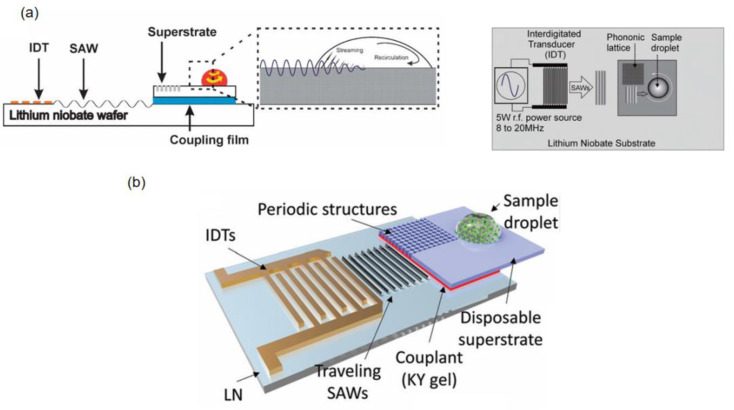
(**a**) Operating principle: travelling SAWs (TSAWs) from IDTs on an LN substrate couple through a thin layer of water and convert to Lamb waves in the silicon superstrate, which are then spatially filtered by an embedded phononic crystal (PnC) for asymmetric droplet actuation. (**b**) Schematic of the acoustofluidic device: a reusable SAW device connects to a disposable superstrate embedded with periodic through-thickness voids (forming a PnC) via a gel couplant. Adapted with permission from [18,53].

The acoustic properties (such as sound velocity and attenuation coefficient) of the coupling agent determine the propagation mode of acoustic waves in microfluidic structures. Ding et al., in their review article, highlight the case of SAW devices assisted by phononic crystals (PnCs), where the coupling agent cooperates with the periodic structure that forms a phononic band gap (Figure 7a [53]), concentrating the sound wave energy in a specific area and achieving frequency-dependent acoustic field focusing. This focusing effect can increase the acoustic energy density by more than 10 times, providing energy support for the directional atomization of droplets and the high-speed aggregation of particles. The same application can also be found in the dual-chip device of Qian et al., which has a silicon overlayer with periodic patterns formed by laser machining at low cost, as shown in Figure 7b [18].

Optimized acoustic impedance matching layers enhance microfluidic manipulation through tailored surface, structural, and material properties. Bourquin et al. [32] used surface-modified agents (e.g., silanized KY gel) to create hydrophobic/hydrophilic patterns for droplet confinement (≤2 mm). Rambach et al. [54] showed that PDMS column geometry controls wave patterns, with 19.5° angled columns enabling directional particle deflection. Key material properties (sound velocity, density) govern wavelength ($\left(\lambda_{f}=c_{f}/f\right)$) and attenuation, requiring precise matching at high frequencies (e.g., 1.1 GHz) [55]. Hard PDMS outperforms soft variants in energy transfer, enabling precise nanoparticle (400 nm) alignment via tilted-angle standing waves [56]. These innovations allow reusable systems by decoupling transducers from disposable microfluidic components while maintaining acoustic precision.

## 4. Key Applications of Acoustic Coupling Agents in Acoustic Flow Control

Acoustic coupling agents play a pivotal role in advancing acoustofluidic technologies across diverse applications, leveraging their unique interfacial properties to enable precise fluid and particle control. In biomedical analysis, their biocompatibility facilitates sensitive cellular manipulations, while biochemical detection benefits from enhanced signal localization. For cell/particle separation, these agents improve sorting efficiency—a critical requirement for diagnostic workflows. Vaporization processes utilize acoustic energy transfer through coupling layers for phase-change applications, whereas droplet manipulation achieves precise screening via synergistic acoustic streaming and thermal effects. Finally, fluid-driven systems exploit optimized impedance matching for energy-efficient transport. Together, these applications demonstrate the versatility of acoustic coupling in modern microfluidic systems. Next, we discuss key applications in acoustic flow control.

### 4.1. High Biocompatibility and Biomedical Analysis

The unique properties of couplants make them indispensable for biomedical applications in acoustofluidics, particularly where biocompatibility and precision manipulation are paramount.

**Figure 8:**
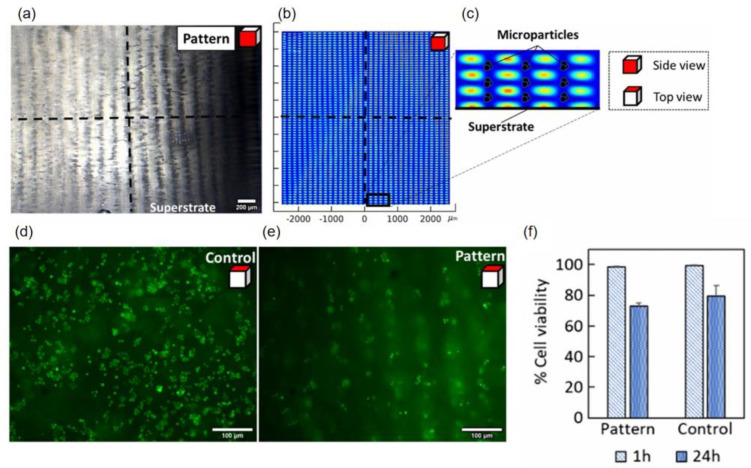
(**a**) Side view of the experimental patterning results (scale bar: 200 μm). (**b**) Simulated acoustic radiation potential distribution. (**c**) Enlarged view of acoustic potential wells showing predicted microparticle trapping locations. (**d**,**e**) Top views of (**d**) control and (**e**) patterned cell samples after staining (scale bar: 100 μm). (**f**) Quantitative cell viability analysis of control and patterned samples at 1 h and 24 h (mean ± standard deviation). Adapted with permission from [34].

Nguyen et al. [34] utilized the acoustic flow platform constructed by an SAW device and selected a 70% ethanol solution as the coupling agent to connect the acoustic flow control platform. This enabled the construction of a micrometer-scale precision 3D particle/cell matrix in collagen hydrogel. The manipulation experiments on 10 μm polystyrene microbeads and MCF-7 cells demonstrated that by adjusting the SAW frequency and power, as shown in Figure 8a, regular patterns with periodic separations of 71 and 159 μm between two nodes could be formed along the vertical and horizontal directions, respectively. Moreover, the pattern remained intact after hydrogel polymerization. The numerical simulation (results shown in Figure 8b,c) was highly consistent with the experimental results, verifying the reliability of acoustic wave transmission in complex media and providing a theoretical basis for large-scale tissue engineering construction. During the experiment, the coupling agent showed good compatibility with collagen hydrogel and MCF-7 tumor cells. The cell viability experiments, as shown in Figure 8d–f, indicated that there was no significant difference in cell viability between the pattern samples and the control samples at 1 h and 24 h (*p*-value > 0.01, *p* = 0.107, with repeated two-factor analysis of variance), which proved that the three-dimensional patterning using SAW devices had no significant effect on cell viability.

**Figure 9:**
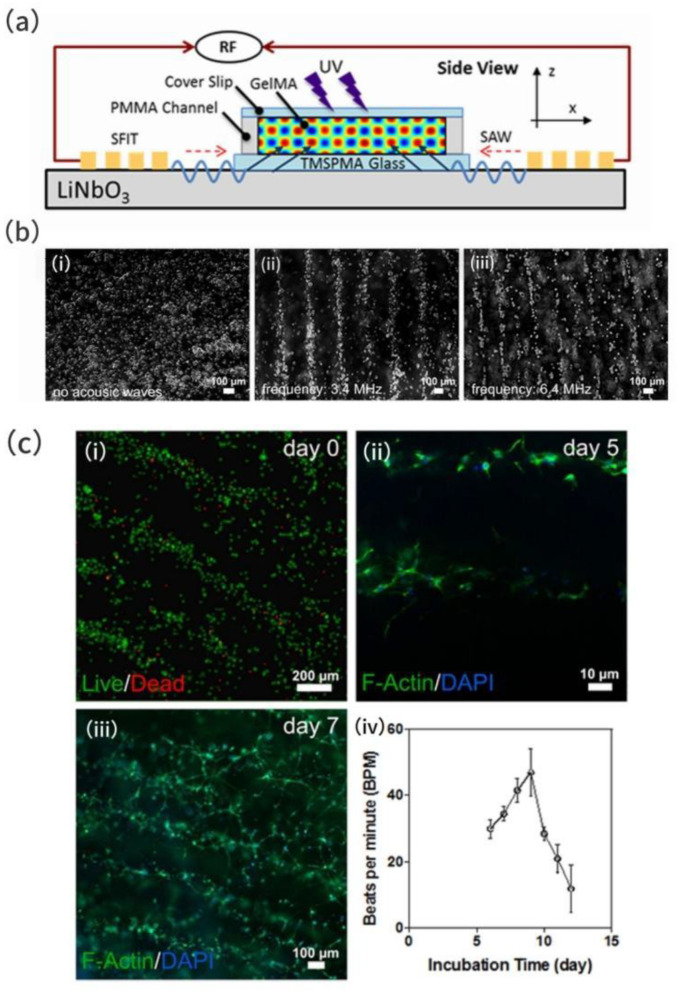
(**a**) Side view schematic of acoustic radiation force patterning. (**b**) Cardiac fibroblast cell patterning: (**b**(**i**)) random cell distribution before acoustic actuation; (**b**(**ii**,**iii**)) aligned cells at (**b**(**ii**)) 3.4 MHz and (**b**(**iii**)) 6.4 MHz after 1.2 s assembly time (scale bars: 100 μm). (**c**) Longitudinal cell viability and functionality: (**c**(**i**)) 90% viability on Day 0; (**c**(**ii**)) F-actin/DAPI staining showing cardiomyocyte dispersion at Day 5; (**c**(**iii**)) interconnected network formation by Day 7; (**c**(**iv**)) spontaneous beating rate evolution of 3D cardiomyocyte tissue. Adapted with permission from [57].

In the experiment conducted by Naseer et al. on the micro-patterned manipulation of cells in hydrogels induced by surface acoustic waves [57], a comb-type transducer (SFIT) on an LN substrate was utilized to generate surface acoustic waves. The acoustic energy was transferred to the cell suspension through a GelMA hydrogel coupling agent and glass, forming standing surface acoustic wave fields (SSAWs), as shown in Figure 9a. The acoustic radiation force (ARF) drives the cells to cluster around the pressure nodes, achieving rapid non-contact patterning (<10 s), and the patterning results are shown in Figure 9b. As GelMA hydrogel serves as the acoustic coupling medium, its viscoelastic properties (density 1020 kg/m^3^, sound velocity 1525 m/s) ensure efficient acoustic wave transmission while providing a biocompatible microenvironment for the cells. As a photo-crosslinkable acoustic coupling agent, GelMA hydrogel is cured by UV irradiation after the acoustic patterning is required, fixing the spatial distribution of the cells. The experiment is shown in Figure 9c. Under short-time UV irradiation (<10 s), the cell survival rate remains above 90%; during long-term culture, the cell morphology is normal and the actin cytoskeleton is clear, indicating that the acoustic manipulation and hydrogel matrix have no significant negative impact on cell function. The patterned cardiomyocytes form an interconnected network within 5–7 days and restore the beating function, and the beating frequency gradually increases with the culture time, indicating that the acoustic manipulation and hydrogel matrix have no significant negative impact on cell function. This proves the feasibility of the acoustic coupling agent technology in constructing functional biological tissues.

In the context of 3D cell patterning for biomedical analytics, 70% ethanol (Nguyen et al. [34]), as an example of liquid-based coupling media, has been shown to enable efficient transmission of surface acoustic waves (SAWs) due to improved acoustic impedance matching. This was used to pattern MCF-7 cells within collagen hydrogels with a periodic accuracy in the range of 71–159 μm, whereby short-term cell viability was similar to the control cohort (*p* > 0.01). Notwithstanding, this coupling medium presents notable limitations. Ethanol exhibits high volatility, necessitating frequent replenishment in prolonged experiments (>30 min); furthermore, elevated concentrations may induce cytotoxicity in sensitive cell types, such as stem cells, thereby precluding its application in long-term culture settings.

Conversely, GelMA hydrogel (Naseer et al. [57]), as an example of gel-type coupling media, possesses viscoelastic properties (density: 1020 kg/m^3^; sound velocity: 1525 m/s) that provide efficient acoustic propagation and a biocompatible microenvironment for cellular populations, with cell viability exceeding 90%. Furthermore, it enables the immobilization of cellular patterns via UV-induced crosslinking. Nonetheless, a critical limitation is that while short-term UV crosslinking (<10 s) exerts minimal deleterious effects on cells, it introduces additional procedural steps. Moreover, post-crosslinking patterns are recalcitrant to reconfiguration, rendering them incompatible with dynamic cell manipulation contexts.

### 4.2. Biochemical Analysis and Detection

Bourquin et al. used a tunable surface acoustic wave (SAW) design [32] to couple acoustic waves through a thin layer of 2 mL KY gel onto a disposable chip (cover layer). KY gel served as the coupling agent that evenly distributes the acoustic energy and works with a heat sink to stabilize the temperature of the droplet to remain below 28 °C, ensuring the activity of biomolecules (such as antibodies, antigens) was not damaged by heat. At the same time, the KY gel had no chemical reaction with disposable chip materials (such as glass coverslips) and biological samples (such as antibody and antigen solutions), maintaining the biological activity of the detection system.

Zhao et al. utilized a one-time acoustic flow control chip constructed with hard and soft PDMS membranes to reduce the energy loss of acoustic waves in the coupling layer due to the low attenuation characteristic of hard PDMS [56]. Meanwhile, the flexibility of the soft PDMS cavity ensured the machinability and biocompatibility of the microchannels. In the article, the chip achieved high-purity separation of *Escherichia coli* (*E. coli*) and human red blood cells (RBCs) through the hard PDMS coupling layer. Flow cytometry showed that the purity of RBCs at the top outlet was 94.0%, and that of E. coli at the bottom outlet was 96.1%. Moreover, the bacterial survival rate remained at 97.5% after separation, demonstrating the low damage of the acoustic coupling agent to biological samples. This performance is comparable to that of permanent bonding devices and solves the problem of insufficient acoustic energy in traditional one-time devices, providing a feasible solution for clinical applications such as pathogen detection and blood purification.

In the domain of biochemical analysis and detection, KY gel and hard PDMS, which represent distinct categories of coupling agents, exhibit divergent performance emphases. KY gel, a gel-based coupling agent, is characterized by low attenuation (0.05 dB/cm·MHz), which facilitates a uniform distribution of acoustic energy. When integrated with heat dissipation configurations, KY gel enables the stabilization of droplet temperatures below 28 °C, thereby preserving the activity of biomolecules, such as antibodies and antigens. However, its limitations include strong viscosity, which induces challenges in chip detachment; residual substances may contaminate subsequent assay samples, and it undergoes mild swelling interactions with certain polymer chips (e.g., PMMA).

In contrast, hard PDMS, a polymeric coupling agent, mitigates acoustic energy loss by virtue of its lower attenuation compared to soft PDMS, achieving high-purity separation of *Escherichia coli* from red blood cells (purity ranging from 94.0% to 96.1%) while maintaining bacterial viability at 97.5%. A critical limitation, however, lies in its lower thermal conductivity. Under high-power conditions (>350 mW), hard PDMS is prone to heat accumulation (with a temperature rise of 7–8 °C), potentially damaging thermosensitive biomolecules such as enzymes.

### 4.3. Cell/Particle Manipulation

The ability to separate particles/cells is of great significance for biological and chemical analysis [10,58].

**Figure 10:**
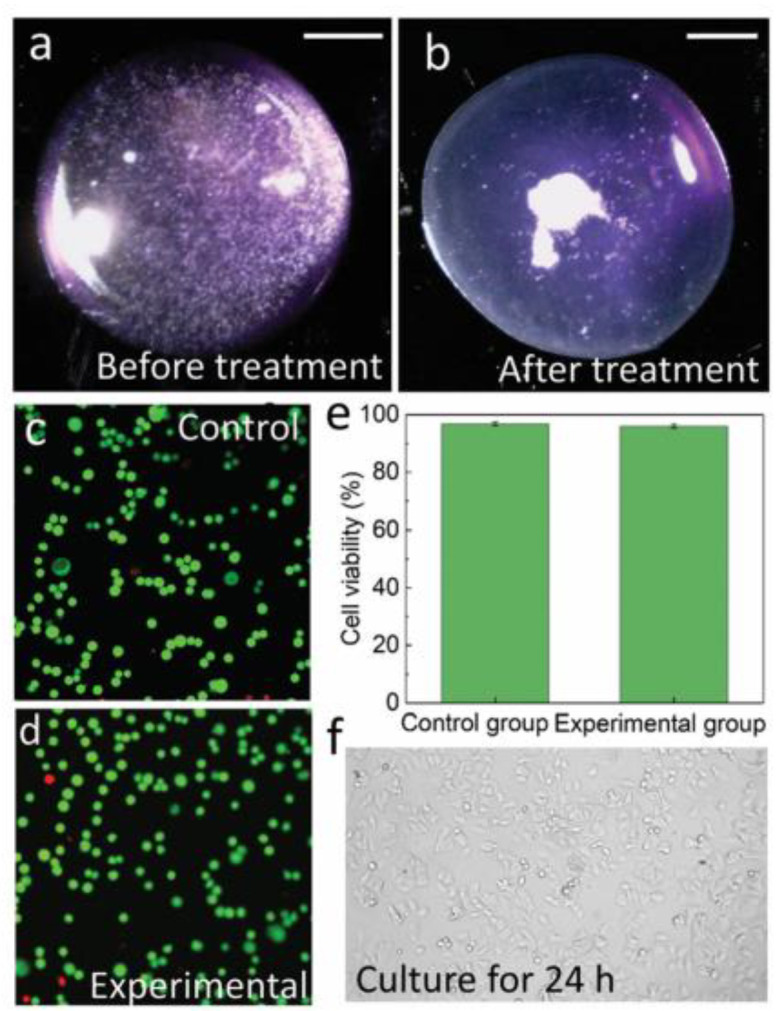
Tumor cell (MCF-7) manipulation and viability assessment. (**a**,**b**) Acoustic focusing of cells in a sessile culture medium droplet. (**c**,**d**) Fluorescence viability testing: (**c**) the control group shows predominantly green fluorescence (live cells) with minimal red fluorescence (dead cells); (**d**) the experimental group (38 dBm, 1 min acoustic treatment) demonstrates no significant increase in red fluorescence. (**e**) Quantitative viability analysis. (**f**) Post-treatment cell morphology after 24 h incubation at 37 °C (scale bar: 1 mm). Adapted with permission from [18].

KY gel, as a biocompatible coupling agent, enabled efficient particle separation and cell viability maintenance in Qian et al.’s acoustofluidic platform [18]. The system achieved density-based separation of PS and silica particles within 30 s of acoustic actuation while maintaining 95.9% cell viability in MCF-7 tumor cells—comparable to controls (96.8%) (Figure 10a–e). Post-treatment cell morphology after 24 h incubation (Figure 10f) confirmed minimal biological impact. The disposable silicon chip design, coupled with the gel’s easy removal, prevented sample device contact, eliminating cross-contamination risks for clinical applications.

**Figure 11:**
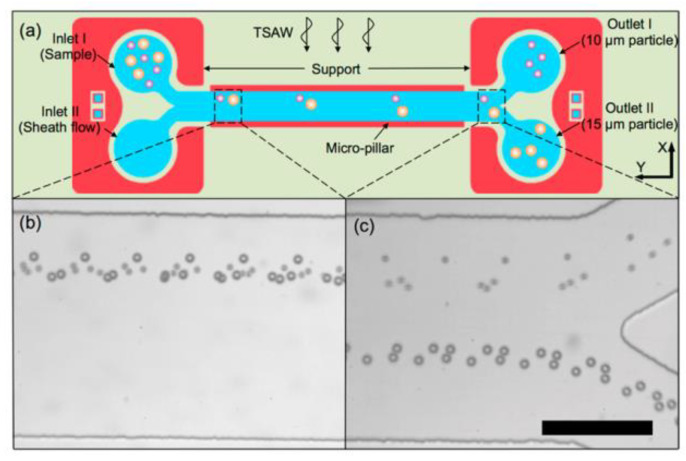
Size-based separation of 10 μm and 15 μm particles using a detachable acoustofluidic system. (**a**) Schematic of the experimental setup. (**b**) Hydrodynamic focusing on the inlet junction: particles are confined near one channel wall by a faster sheath flow. (**c**) Tilted-angle standing surface acoustic wave (TSAW, 49.5 MHz) separation: larger particles (15 μm) deflect toward Outlet II, while smaller particles (10 μm) exit via Outlet I. Adapted with permission from [17].

Ma et al. [17] developed an innovative detachable acoustic flow control system that directly contacts the LN SAW transducer through a PDMS microcolumn structure (600 μm wide × 50 μm thick), without the need for traditional acoustic coupling agents. In this design, the microcolumn serves as a bridge for acoustic energy conduction. Through Rayleigh angle refraction ($\theta_{R}^{PDMS}\approx 14.8{}^{\circ},\theta_{R}^{water}\approx 22.1{}^{\circ}$), the sound waves are efficiently introduced into the fluid channel, significantly reducing the energy loss at the interface. Experiments show that, driven by 49.5 MHz traveling wave surface acoustic waves, this system can efficiently separate 15 μm and 10 μm particles (efficiency > 98%). The unique microcolumn structure generates a unidirectional traveling wave field, enabling the particles to continuously move along the direction of wave propagation and overcoming the problem of limited displacement in the standing wave field. This point-contact design enables the PDMS channel module to be easily disassembled and replaced, while the transducer can be reused. This not only meets the demand for disposable consumables in biomedical testing but also effectively avoids the cross-contamination of biohazard samples while maintaining excellent biocompatibility. The particle separation process and effect of the entire system are detailed in Figure 11a–c.

In the realm of cell and particle manipulation applications, the high stability of KY gel (Qian et al. [18]) provides consistent coupling efficiency over 25 min. KY gel, as a couplant, not only enables density-based separation of polystyrene particles and silica particles within 30 s but also ensures a survival rate of 95.9% for MCF-7 cells (approaching the 96.8% observed in the control group). However, its limitations include strong adhesiveness to silicon chips, requiring ethanol cleaning when separating the chip from the SAW transducer and thus interfering with cell viability. Additionally, acoustic attenuation increases when the gel thickness is more than 30 μm.

In contrast, PDMS microcolumn structures (Ma et al. [17]), which obviate the need for conventional coupling agents, directly conduct acoustic energy through microcolumns that are 600 μm wide and 50 μm tall. These microcolumn structures enable a separation efficiency exceeding 98% for 10 μm and 15 μm particles while circumventing the issue of coupling agent contamination. A critical constraint, nonetheless, is that such microcolumns require extremely high fabrication precision (with tolerances below 5 μm) to avoid non-uniform acoustic energy distribution. Moreover, such an approach is only applicable to particle sizes within a specific range (10–15 μm).

### 4.4. Atomization

Atomization is the process of transforming small solid particles or droplets into aerosols. Kurosawa et al. first proposed and constructed a novel ultrasonic atomizer using a TSAW device [59]. Since then, atomization based on SAW devices has been widely applied [59,60,61].

**Figure 12:**
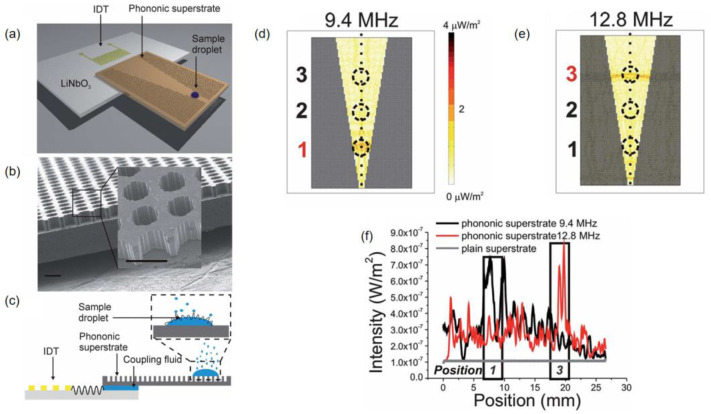
(**a**) Schematic of the acoustofluidic device comprising an LN substrate with IDTs and a PnC cone superstrate. (**b**) PnC lattice design: hexagonal array of holes (diameter = 160 μm, pitch = 200 μm, depth = 235 μm) etched in silicon (scale bar: 200 μm). (**c**) Cross-sectional operation mechanism: IDT-generated SAWs couple into the disposable superstrate as Lamb waves, which are focused by the PnC lattice to create capillary waves that induce droplet pinch-off. (**d**,**e**) Droplet formation patterns on the PnC superstrate at (**d**) 9.4 MHz and (**e**) 12.8 MHz (droplet positions marked by dashed circles). (**f**) Acoustic intensity profile across the superstrate (along dotted line in **d**,**e**) showing enhanced focusing at PnC frequencies (9.4/12.8 MHz) versus plain superstrate, with droplet Positions 1 and 3 highlighted. Adapted with permission from [31].

Reboud et al. [31] demonstrated a PnC-enhanced atomization system using KY gel as a coupling agent (5 mL) to achieve efficient acoustic energy transfer between the piezoelectric substrate and disposable cover layer (Figure 12a,c). The gel’s high impedance matching minimized interfacial reflections, enabling effective Rayleigh-to-Lamb wave conversion for droplet atomization. Unlike conventional methods relying solely on coupling agents, this design synergized the gel with a PnC-patterned cover (Figure 12b), preventing energy dispersion and reducing atomization time. The structured cover compensated for acoustic losses inherent in flat interfaces, showcasing a coordinated approach to optimize both wave transmission and spatial energy focusing.

The integration of acoustic interface materials with PnC superstructures boosts atomization efficiency by 50% compared to conventional silicon substrates, achieved through bandgap-controlled acoustic focusing that induces rapid liquid interface destabilization via capillary stress. This mechanical-actuation-free system relies solely on frequency-tuned SAW excitation to generate micron-sized droplets. Frequency modulation (12.8 MHz vs. 9.4 MHz) enables selective droplet processing at different superstructure locations (Position 3 entrance vs. Position 1 vertex, Figure 12d–f), creating a contactless, high-throughput platform ideal for mass spectrometry sample preparation where site-specific atomization and contamination prevention are crucial.

Acoustic coupling agents enable efficient energy transfer between reusable LN substrates and disposable superstructures, significantly reducing costs while maintaining performance. The superstructure features a photolithography-patterned square lattice (160 μm apertures, 200 μm spacing) with hydrophobic/hydrophilic treatment (106.4 ± 1.0° contact angle) to precisely position droplets within 2 mm hydrophilic zones. KY gel coupling ensures optimal impedance matching for acoustic transmission, allowing stable handling of 0.5–2 μL droplets—ideal for drug delivery and point-of-care testing (POCT) applications requiring small-volume precision. This approach eliminates expensive piezoelectric material processing while meeting microfluidics’ “small volume, high precision” demands through mass-producible disposable components.

### 4.5. Droplet Manipulation

The ability to precisely select a desired droplet from among other droplets and manipulate the droplet’s properties is crucial for chemical and biological screening. It has been demonstrated that the acoustic streaming induced by surface acoustic waves (SAWs) can be used to manipulate droplets, aided by coupling agents.

**Figure 13:**
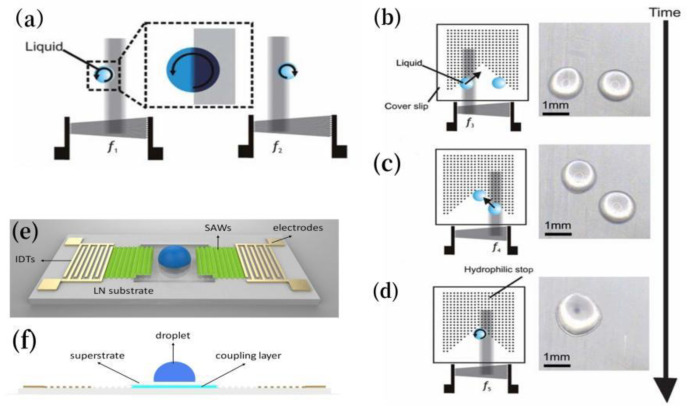
(**a**) Acoustic streaming control: anti-clockwise (9.6 MHz) and clockwise (11 MHz) vortices generated by frequency-modulated SAWs. (**b**–**d**) Device schematics with sequential fluid operations: (**b**) droplet transport, (**c**) merging/mixing, and (**d**) silver nanoparticle concentration via acoustic centrifugation (insets: experimental snapshots). (**e**) Overview of the acoustofluidic platform architecture. (**f**) Cross-sectional schematics detailing layer-by-layer integration. Adapted with permission from [50,62].

In the integrated microfluidic system of tunable surface acoustic waves for liquid and particle manipulation on a single chip developed by Bourquin et al. [62], the surface acoustic waves propagate in the piezoelectric wafer in the form of Rayleigh waves, which then couple into the one-time cover layer through a thin layer of aqueous solution (e.g., water or saline solution), forming Lamb waves in the glass cover slip. This coupling mechanism lays the foundation for the application of “lab-on-a-chip” technology in low-cost and immediate diagnosis. By adjusting the excitation frequency (such as $f_{3}=11\;MHz$, $f_{4}=9.2\;MHz$, and $f_{5}=9.6\;MHz$), droplets can move along the hydrophilic point track and merge, as shown in Figure 13b–d. The schematic diagram shows the counterclockwise and clockwise flows (Chebyshev flows) generated by the SAWs at frequencies $f_{1}\approx 9.6\;MHz$ and $f_{2}\approx 11\;MHz$, as shown in Figure 13a, which can achieve reagent mixing; by breaking the symmetry of the SAWs and inducing particles to accumulate towards the center of the droplet, centrifugal concentration is completed (such as efficient enrichment of 10 μm  polystyrene beads). The entire process from basic fluid manipulation to chemical reactions is demonstrated.

Xu et al. developed an acoustofluidic system for synthesizing hierarchical porous metal–organic frameworks (MOFs) using asymmetric surface acoustic waves (SAWs). As shown in Figure 13e,f, the system consists of an LN substrate with IDTs, a replaceable/“renewable” coupling layer, and a disposable glass cover. The coupling layer converts the substrate’s linear SAWs into nonlinear Lamb waves, which propagate asymmetrically in the cover layer with sufficient intensity to generate strong acoustic streaming in droplets. This acoustic agitation enhances solute transport through vigorous mixing, significantly improving ion collision probability. In Cu-BTC synthesis, the acoustic-assisted system achieved complete crystallization within 4 min (9.3 MHz, 10 V), outperforming conventional static methods that yielded incomplete crystals with irregular morphology.

In the applications of atomization and droplet manipulation, the combination of KY gel and PnCs (Reboud et al. [31]) demonstrates distinct advantages. For example, the combination of the gel’s excellent acoustic impedance matching property (1.45–1.60 MRayl) and the focusing ability of the PnC enhances the atomization efficiency by 50% compared to conventional silicon substrates. The PnC also enables precise control of atomization positions through frequency adjustment (9.4 MHz and 12.8 MHz). Yet, its limitations are prominent: PnCs require the fabrication of 160 μm pore arrays via photolithography (i.e., microfabrication). Moreover, KY gel exhibits increased attenuation at higher frequencies (>15 MHz), which is prone to inducing non-uniform atomization.

In contrast, water or saline, as examples of liquid coupling agents (Bourquin et al. [62]), can realize the conversion of Rayleigh waves to Lamb waves by just a thin layer (<2 μm), supporting operations such as droplet transport, fusion, and enrichment of silver nanoparticles. As for drawbacks, within 1 h, their coupling efficiency decreased by 75% due to evaporation, necessitating frequent replenishment. Furthermore, their low viscosity tends to cause droplet deviation.

### 4.6. Fluid-Driven Micromotors

**Figure 14:**
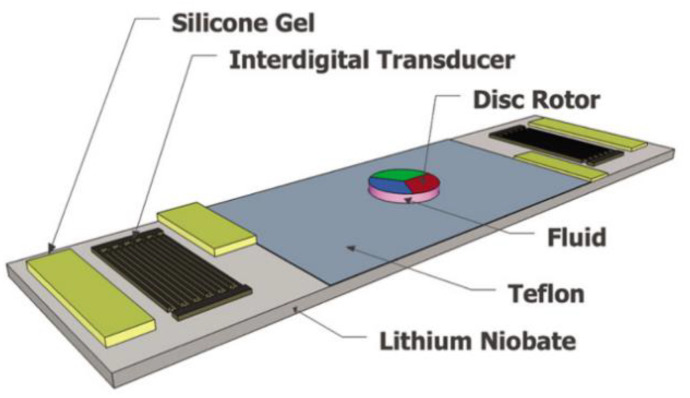
Isometric device schematic. Symmetry-breaking acoustic wave manipulation was achieved by blocking opposite SAW halves from each IDT using silicone gel damping layers. This asymmetric wave coupling into the fluid layer generated controlled rotational motion, with directionality indicated by the bold arrow. Adapted with permission from [63].

Shilton et al. [63] developed a contactless micromotor system using surface acoustic waves (SAWs) and fluid coupling (water/silicone gel) to achieve precise microscale actuation (Figure 14). The design features opposing IDTs on an LN substrate with silicone gel wave absorbers, creating asymmetric SAW propagation that induces rotational flow. A 5-mm rotor achieved 2250 rpm (139 m/s^2^ radial acceleration) through viscous coupling, enabling functions like particle concentration and centrifugal separation. Compared to the electrowetting deformation-based micromotor reported by Takei et al. [64] (maximum rotational speed < 1000 rpm), Hilton’s work demonstrates an order of magnitude improvement in rotational speed.

This micromotor system can be integrated with microfluidic chips. Through dynamic adjustment of the surface acoustic wave frequency and amplitude, such a micromotor-integrated microfluidic device can produce coordinated operations of various functions, such as fluid mixing, particle sorting, and centrifugal sequencing. Its compact design (without the need for large auxiliary equipment) makes it suitable for portable point-of-care detection devices. The application of the coupling agent in this new design opens new ideas for the application of acoustic coupling agents in future acoustic flow control.

In the application of fluid-driven micromotors, a hybrid system couplant composed of water and silicone gel (Shilton et al. [63]) was used to induce rotational flow via asymmetric surface acoustic waves (SAWs) to deliver remarkable driving performance, a 5 mm rotor reaching a rotational speed of 2250 rpm, far exceeding the upper limit of 1000 rpm for electrowetting motors. However, this system has obvious limitations: the liquid coupling agent is prone to splashing at high rotational speeds. Moreover, the high attenuation property of silicone gel results in an energy conversion efficiency of only 30%, which is significantly lower than PDMS (50%), excluding its application in scenarios demanding high precision and low loss.

## 5. Technical Challenges and Optimization Strategies

The current challenges in the application of various types of acoustic coupling agents in acoustic flow control-related devices include the stability of the coupling agents, the influence of the coupling agents on acoustic energy transmission, the compatibility between the target layer, SAW devices, and coupling agents, etc. Next, the technical challenges mentioned above will be elaborated and the existing relevant optimization methods will be presented.

### 5.1. Stability of the Coupling Agent

#### 5.1.1. Evaporation and Degradation of the Liquid Coupling Agent

The problem of evaporation and degradation of liquid coupling agents becomes prominent under high RF power application. Adding oil to water (as the coupling layer) can reduce evaporation during long-term operation, but the increase in viscosity may also affect the experimental results [8]. More commonly, a suitable acoustic coupling gel is selected to alleviate the effects, such as evaporation [47]. Currently, there have been studies on the acoustic properties of liquid coupling agents containing viscous substances. However, their actual performance in acoustofluidic devices, particularly in terms of energy transmission efficiency and long-term stability, remains to be systematically evaluated through controlled experiments. For short-term and low-power acoustic energy experiments, the convenience brought by ease of use and preparation, rather than long-term stability (evaporation and degradation), is often of more urgent concern practically. Adding appropriate porous polymer materials to the liquid coupling layer to control the evaporation rate of the liquid, using automated equipment to monitor and obtain the dissipation information of the coupling layer, and timely supplements and adjustments are also possible directions for future experiments.

#### 5.1.2. Thickness Control of Liquid Couplants

The thickness of liquid couplants (e.g., water or glycerol) is difficult to control precisely during device operation, often resulting in unstable positioning of the covering layer due to fluid flow or evaporation. Using double-sided adhesive tape separators [36] and metal clamps [65] can reduce the movement of the liquid coupling layer. But, mechanical clamping and adhesion inevitably introduce additional damping and reduce the transmission efficiency of acoustic waves. The moving characteristics of the liquid coupling agent are its inherent physical properties. Whether liquid coupling layers with different thicknesses and different additives are effective in stabilizing the couplant has yet to be validated. The common practice so far has been to choose coupling agents that are more stationary to replace liquid coupling agents. PDMS films with excellent mechanical properties and UV epoxy resin’s photo-sensitive characteristics are often selected in experimental methods as suitable materials to replace liquid coupling agents.

### 5.2. The Influence of Coupling Agents on Acoustic Energy Transmission

#### 5.2.1. Thermal Management

The acoustic impedance and attenuation coefficient of the coupling layer will cause a slight drop in amplitude within the transmission frequency band of the acoustic wave. The heat generated in the overlaying layer is coupled with the energy of the interfacial polymer layer. The higher the energy coupling efficiency of the polymer film, the higher the proportion of surface acoustic wave energy on the LN SAW substrate that leaks into the superstrate microfluidic chip in the form of Lamb waves, which ultimately converts into thermal energy. Discrete operation periods can be adopted to limit the increase in temperature [20]. In the case of SAW devices, a variable duty cycle acoustic wave generator is used to cope with the adverse effects of temperature rise. Another approach is adding cooling channels to counteract the thermal rise. With the aim of improving cell viability, the above methods provide interesting research directions for further investigation.

#### 5.2.2. Acoustic Coupling Multilayer Synthesis

Kolesnik et al. [47] suggest that to maximize transmission efficiency, the relative thicknesses of the superstate layer and the coupling layer must be optimized simultaneously. This optimization can achieve similar maximum transmission efficiency across various operational conditions. They found that although the thickness of the coupling layer does indeed affect the transmission of acoustic waves, the optimal size is approximately one quarter [47]. When the precise thickness of the coupling layer is difficult to control, focusing on the relation between the cover layer and the coupling layer may provide practical alternatives for a reasonable thickness. For acoustic flow control applications, such as droplet operation and particle control, the movement of particles is strongly related to their own diameter and acoustic energy efficiency, and the uniformity and stability of acoustic energy transmission are crucial. The combined use and coordinated cooperation of different coupling layers can achieve the maximum stability in sound energy transmission. To date, multilayer coupling agents have been applied in the form of liquid coupling layers and PDMS channels [52] and double-layer PDMS films to combine the characteristics of different coupling agents. The multilayer approach aims to utilize the advantages of multiple combinations while reducing the influence of the constituent layer’s drawbacks (such as instability, high damping coefficient, and rapid evaporation).

### 5.3. Compatibility Between the Target Layer, SAW Devices, and Coupling Agents

Water, the simplest coupling agent, offers wide applicability with no restrictions on the target object. When choosing a polymer coupling agent, its compatibility with the surrounding covering layer, piezoelectric sound-generating plate, and other devices must be carefully considered.

To fit the size of SAW devices and upper microchannels, PDMS can be shaped using extrusion, soft lithography, pressure lithography, coating, and molding techniques. The viscoelasticity of the polymer can be adjusted by crosslinking and adding fillers, like silica, to the polymer network. In experiments, thin PDMS coupling layers are often required. Unlike soft PDMS, hard PDMS has a lower acoustic attenuation coefficient and can be made into ultrathin films, increasing the sound pressure in the microchannel.

Currently, PDMS film research in acoustic flow control mainly focuses on SAW, with little attention on the combination of bulk acoustic waves and PDMS films. Coupling layers connect acoustic wave transmission, substrates, and target layers, and their connection between the layers is vital. Studying the compatibility of different layers is challenging and will require multiple experiments. Different composition ratios of polymer materials lead to various characteristics. For example, UV epoxy resin can be liquid or solid under ultraviolet light, which can be used for sound energy transmission, device connection, and post-experiment processing, ensuring efficient experiments and promoting the development of disposable and reusable devices.

### 5.4. Future Perspectives and Optimization Strategies

The acoustic transmission characteristics (e.g., acoustic impedance, attenuation coefficient) of conventional liquid-based, gel-based, and polymer-based couplers employed in current acoustofluidic platforms exhibit significant dependence on material thickness, volume, and physical state (liquid/gel phase). Their properties are highly susceptible to temperature fluctuations and acoustic frequency variations, often resulting in experimental data inconsistencies or device performance instability. Recent advances in smart responsive couplers, multilayer architectures, and hybrid technologies have provided innovative solutions towards addressing this issue.

**Smart Responsive Couplers:**

Thermoresponsive couplers (e.g., PNIPAM-based hydrogels), as reviewed by Guleria et al. [66], utilize temperature-dependent properties to autonomously regulate acoustic attenuation. Polymer chain contraction at elevated temperatures reduces acoustic energy dissipation, while expanded structures at lower temperatures enhance wave scattering. This temperature-controlled state switching eliminates reliance on external stimuli (e.g., UV light), thereby simplifying experimental procedures. Photo-curable couplers (e.g., UV gels) form stable solid interfaces through UV-initiated in situ polymerization, preventing acoustic impedance mismatch caused by fluid flow. Future developments may focus on visible-light-curable materials with lower energy requirements to minimize UV-induced damage to biological samples. Field-responsive couplers can modulate their morphology or internal structure in response to external electric or magnetic fields [67]. For instance, magnetic fluids enable precise control of acoustic wave propagation paths (e.g., focusing or beam steering) through field-induced nanoparticle alignment, while electrorheological materials permit real-time viscoelasticity adjustment via applied electric fields to achieve frequency-specific impedance matching.

**Multilayer Coupling Architectures:**

Optimized hierarchical structures are crucial for synergistically combining material advantages (e.g., low attenuation of liquids, stability of gels, and reusability of polymers). A liquid gel hybrid bilayer design, incorporating a thin aqueous layer (low attenuation) and a top gel layer (evaporation-resistant), has demonstrated a 300% extension in operational duration compared to single-liquid-layer systems [36]. This design, validated in high-power surface acoustic wave devices [44], effectively reduces energy loss and prevents droplet displacement. PDMS-based multilayer structures combining rigid PDMS (low attenuation) with soft PDMS (flexibility) enhance energy transfer to silicon substrates while maintaining interfacial conformity [49]. Kolesnik et al. [47] demonstrated that an optimized layer thickness ratio (coupling layer to capping layer of 1:4) provides >90% transmission efficiency across a wide operating frequency range of 10–50 MHz, which is critical for broadband acoustofluidic applications.

**Integration with Advanced Acoustofluidic Systems:**

In terms of system integration, PnC couplant hybrids combining KY gel with PnC overlayers [31] can enhance acoustic focusing tenfold, enabling targeted nebulization and high-throughput mass spectrometry sample preparation. Future designs may incorporate reconfigurable phononic lattices for dynamic field shaping. Artificial intelligence-driven optimization employing machine learning models trained on acoustic property datasets (impedance, attenuation) has been used to synthesize atypical compositions for specific applications to reach 94% accuracy in cell sorting applications [68]. This approach significantly accelerates material screening and addresses current limitations based on trial-and-error methodologies.

## 6. Conclusions

Acoustic coupling agents serve dual roles in energy transfer and interface adaptation for acoustofluidic systems. Their material properties critically determine device performance, with different types offering distinct advantages: liquid agents (e.g., ethanol) enable low-loss bioanalysis, gel-based materials (e.g., KY gel) provide tunable viscoelasticity for stable yet reversible bonding, and PDMS films allow reusable van der Waals interfaces. These agents precisely control wave modes (e.g., Lamb waves) and acoustic fields, enabling efficient particle manipulation, droplet sorting, and cell handling. However, challenges persist regarding evaporation, thermal effects, and multilayer impedance matching. Emerging solutions include nanocomposite gels (e.g., SiO_2_/agarose), hybrid PDMS liquid systems, active cooling, and optimized impedance designs. Future development should focus on long-term stability in biological environments and smart material integration (e.g., photo-responsive gels) for dynamic field control—advances that will accelerate clinical translation in tissue engineering and precision medicine.

## Figures and Tables

**Table 1:** Comparison of properties between water and 100 cSt silicone oil.

Parameter	Aqueous (Water)	Oil-Based (Silicone Oil 100 cSt)
**Acoustic Impedance**	1.483 MRayl (20 °C)	0.95 MRayl
**Sound Velocity**	1.48 mm/μs (20 °C)	0.98 mm/μs
**Density**	1.00 g/m^3^	0.968 g/m^3^
**Temp. Sensitivity**	High (*γ* = +2.4)	Low

(Data source: Onda Corporation, 2003 [42]).

**Table 2:** Acoustic impedance and properties of selected solid materials.

Material	Type	VL (mm/μs)	VS (mm/μs)	Density (g/cm^3^)	ZL (MRayl)	F	Loss (dB/cm)
**DER317 Epoxy (9phr DEH20, 110phr W)**	Epoxy resin	2.18	0.96	2.04	4.45	–	6.6 @ 2 MHz
**Stycast 1264 (45phr, 600phr W)**	Epoxy resin	1.65	–	4.71	7.77	–	29.7 @ 5 MHz
**Silver Epoxy (E-Solder 3022)**	Conductive adhesive	1.90	0.98	2.71	5.14	–	16 @ 2 MHz

V_L_ denotes the longitudinal wave velocity, while V_S_ denotes the shear wave velocity. (Data source: Onda Corporation, 2003 [46]).

## Data Availability

No new data were created or analyzed in this study.
